# Malignancy-related mir-210, mir-373 and let-7 levels are affected in iron deficiency anemia

**DOI:** 10.4314/ahs.v23i3.30

**Published:** 2023-09

**Authors:** Ruveyda Sak, Demircan Ozbalci, Emine Guchan Alanoglu, Kuyas Hekimler Ozturk

**Affiliations:** 1 City Hospital of Etlik, Department of Rheumatology; 2 Suleyman Demirel University School of Medicine, Department of Hematology; 3 Suleyman Demirel University School of Medicine, Department of Medical Genetics

**Keywords:** miRNA, iron deficiency anemia, malignancy

## Abstract

**Background:**

Hypoxia is the hallmark of iron deficiency anemia (IDA) and in hypoxic environment, significant changes are observed in malignancy-related microRNAs (miRNA). Our aim is to examine whether there is any difference in the levels of miR-210, miR-373 and let-7, which are directly related to malignancies in patients with IDA.

**Methods:**

Thirty-five female patients with IDA between the ages of 18-65 and 10 healthy controls were included in the study. Patients who received oral iron therapy, who had inflammatory disease, and who were pregnant were excluded from the study. Student t Test was used for comparing variables with normal distribution in two independent groups, and Mann-Whitney U Test was used for variables without normal distribution. Comparison of categorical data was made using the chi-square test.

**Results:**

The mean hemoglobin and ferritin level were 10,78±0,93 and 6.28±5,76 respectively. Plasma miR-210 expression were found as -7.27±2.23 and -6.15±0,88 in IDA and control group respectively (p = 0.022). Plasma miRNA-373 were -7.36±2,58 and -6,96±1,93 and let-7 expression were 2.14±2,15 and 3,57±2,21 in IDA and control group. (p = 0.65 and p = 0.20, respectively).

**Conclusions:**

Plasma miR-210 expression was significantly up-regulated and miR-373 and let-7 expression was down-regulated, though insignificantly, in IDA group.

## Introduction

Iron deficiency anemia (IDA) is the most common type of anemia in the world. It has negative effects such as workforce losses and impairing the quality of life, cognitive, sexual and the immune system dysfunction 1. Malignancies also especially play an important role in the etiology of IDA in elderly, which is easy to diagnose and treat^2^. IDA is linked to oxidative stress and oxidative stress is associated with mitochondrial and genomic instability which may lead to tumour angiogenesis and metastasis and inhibit apoptosis 1. In addition, it is thought that IDA disrupts the activity of natural killer cells and lymphocytes and, blocks functioning of peroxidase, which may lead to infections and malignancies 3, 4. Iron is also a cofactor of catalase which leads to lower catalase and higher oxidative stress in IDA 5. Regarding genomic instability, iron is a co-factor in DNA helicase and ribonucleotide reductase. DNA repair enzymes need iron-sulphur cluster component for recognizing DNA damage 6. Also, the m2 sub-unit of ribonucleotide reductase uses iron as a cofactor and malfunctioning of ribonucleotide reductase was linked to mutation and cell death 7. Although there was theorical evidence that iron deficiency might be linked to carcinogenesis; there were few studies addressing the problem. Berrak et al, had found that children with iron deficiency had decreased rate of apoptosis. The degree of apoptosis deficiency was negatively correlated with degree of iron deficiency and iron supplementation was found to correct apoptosis rate 8. Wen et al showed that maternal oral iron medications significantly decreased the incidence of childhood acute lymphoblastic leukemia 9. Low iron intake also might be responsible for development of colorectal cancer in older patients 10.

MicroRNAs (miRNA) are small (about 22 nucleotides in length), single stranded fragments of non-coding RNA. Recently, it has been determined that these particles, which are thought to be non-functional, have very important functions in protein transcription and translation and are effective in the development of many diseases, especially malignancies 11-14. After hypoxia, it has been shown that, activation of miR-210 and miR-373 reduces gene expressions responsible for DNA repair 14. Let-7 is a tumour suppressor miRNA that is associated with tumour suppression and down-regulation of this miRNA was associated with tumour progression 15. IDA is clearly associated with tissue hypoxia, and it was speculated that, the expression of genes responsible for DNA repair and tumour suppression might be downregulated in IDA related hypoxia 15. Since, IDA is more prevalent in females, our aim is to examine whether there is any difference in expressions of miR-210, miR-373 and let7 in female patients with IDA.

## Methods

This case-control, non-randomised study was approved by the local Ethics Committee of Suleyman Demirel University on December 13^th^, 2018, with number 238. All patients had given written consent about the study. Thirty-five female patients with IDA between the ages of 18-65 who applied to our outpatient clinic and ten healthy individuals who did not use any medication as the control group were included in the study. Most patients were selected in city of Isparta; populated by 445678 inhabitants, has an altitude of 1050 meter, and located in the southwest part of Turkiye. 22 % of patients came from Dinar; a town of Afyonkarahisar city and Burdur city located 63 and 30 km away from Isparta respectively. Patients' selection was on a non-random basis; patients diagnosed as IDA in our outpatient clinic and had agreed to participate were included in the study. Patients who received oral iron therapy, who had inflammatory disease (acute infectious diseases, malignancies, rheumatic diseases), who had leukopenia or thrombocytopenia and who were pregnant were excluded from the study. IDA was defined in patients as hemoglobin levels below 12 gr/dl and ferritin counts below 20 U/fl.

Complete Blood Count analysis was performed on the Beckman Coulter DxH800 (Brea, CA, USA) device using the Coulter method. Hemoglobin was measured by photometric method at 525 nm and hematocrit, mean corpuscular haemoglobin and, mean corpuscular haemoglobin concentrations were additionally calculated. Mean corpuscular volume and red cell distribution width obtained from red blood cell histogram. Ferritin was studied in the Cobas e 601 (Mannheim, Germany) device using the electrochemiluminescence immunoassay (ECLIA) method.

For evaluating miRNA concentration of patients and control group, 4 cc of blood was drawn from patients to EDTA tubes. The collected blood was rotated for 10 minutes at 15,000 rpm in a cooled centrifuge within 1-2 hours, and the plasma part was stored at -800C until the cDNA isolation. Total miRNA isolation was performed using the Hybrid-RTM miRNA Isolation Kit (GeneAll Biotechnology, Korea) in accordance with the manufacturer's instructions and the measurements of the isolated total miRNAs were made. Samples with A260/280 ratio below 1.8 or A260/230 ratio below 2.0 were not included in the study. From the obtained total miRNA, cDNA was obtained using the WizScript™ cDNA Synthesis Kit. RT-PCR steps were performed in accordance with the manufacturer's instructions, using the StepOnePlus Real Time PCR (Thermo Fisher Scientific, US) device. Selected miRNAs were normalized using the RNU6B housekeeping gene. The intergroup fold change of miRNA levels was done using the 2-ΔΔCt equation. The primary sequences of the miRNAs we investigated were, F 5′-AGCCCCTGCCCACCGCACACTG for miR-210, 5′-GAAGTGCTTCGATTTTGGGGTGT for miR-373, 5′- CTGTACAACCTTCTAGCTTTCC for let-7.

Student t Test was used for comparing variables with normal distribution in two independent groups, and Mann-Whitney U Test was used for variables without normal distribution. Comparison of categorical data was made using the chi-square test (gender). The independent effect of miRNAs on the development of DEA was evaluated by logistic regression analysis. SPSS for Windows version 22.0 package program was used for statistical analysis and p <0.05 was considered statistically significant.

## Results

In our study, a total of 45 patients, 35 patients with IDA and 10 healthy controls were included. 60 % of the study group were living in city centres and 40 % were living in rural areas. All participants in control group were living in Isparta city centre, they all were graduated from university and had a job. On the other hand, in patient group, 54 % were living in Isparta (of them, 47% in rural area) and, only 31 % had a job. In patient group 14 % was graduated from primary school, 23 % from secondary, 49 % from high school and only 14 % were graduated from university. All study groups were female and 57.1% (n = 20) had comorbid diseases (one had hypertension, one had coronary artery disease, two had asthma (one also had thyroid disease), three had diabetes, eleven had thyroid diseases, three had other comorbidities). The ages showed a homogeneous distribution and no statistically significant difference was found between the groups (p = 0.063). In the control group, hemoglobin, hematocrit, white blood cell count, neutrophil count, mean corpuscular volume and ferritin values were significantly higher than the IDA group, while the red cell distribution width value was significantly lower (p <0.05) (Table 1). There was no significant difference between the control group and the patient group in terms of lymphocyte count, platelet count and mean platelet volume.

**Table 1:** Age, total blood count and ferritin levels of groups

	Control	Patient	*p* value
**Age**	31,3±6,25	38.46±11.29	0.063
**Age groups**			
**10-20**	-	3	
**20-30**	5	5	
**30-40**	4	8	
**40-50**	1	15	
**50-60**	-	4	
**Total**	10	35	
**WBC**	8,25±2.45	6.43±1.44	**0.048**
**NEU**	5,14±1.75	3.81 ±1.08	**0.005**
**LYN**	2.23±0.84	1.94 ±0.50	0.191
HB*	14,0±0,77	10,78±0,93	***p*< 0.001**
HCT*	40,39±2,33	33.27±2.27	***p*< 0.001**
**MCV**	84,98±3,40	73.22±7.33	***p*< 0.001**
**RDW**	13.62±0.79	16.60±1.75	***p*< 0.001**
**PLT**	270,30±65,12	309,4±76,27	0,148
**MPV**	8,78±0,87	8.39±0.93	0,25
FERRİTİN*	24,15±13,51	6.28±5,76	***p<*** **0.001**

Data are given as mean±SS and median±ÇAA*. Leukocyte WBC; Neutrophil, NEU; Lymphocyte, LYN; Hemoglobin, HB; Hematocrit, HCT; Mean Erythrocyte Volume, MCV; Distribution Width by Size of Erythrocytes, RDW; Thrombocyte, PLT; Mean Platelet Volume, MPV

**Figure 1:**
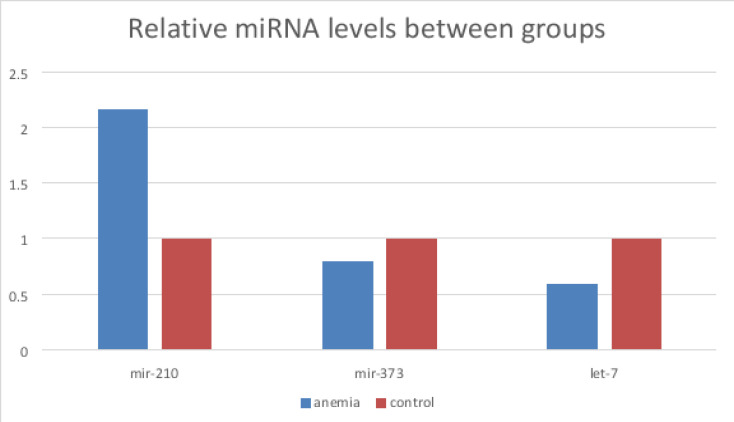
Relative MiRNA levels between groups

Plasma miR-210 expression was significantly upregulated in the IDA group compared to the control group (p = 0.022). Plasma miR-373 and let-7 expression were found to be downregulated in the IDA group compared to the healthy controls but, no significant difference was found between the groups (p = 0.65, p = 0.20, respectively) (Table 2 and Figure 1).

**Table 2:** Plasma miRNA levels of groups

		N	Mean±SD	*p* value
**MiRNA-210ΔCt**	**Patient**	35	-7.27±2.23	**0.022**
	**Control**	10	-6.15±0,88	
**MiRNA-373ΔCt**	**Patient**	35	-7.36±2,58	0.65
	**Control**	10	-6,96±1,93	
		**N**	**Median±CAA**	*p* **value**
**Let-7ΔCt**	**Patient**	35	2.14±2,15	0.20
	**Control**	10	3,57±2,21	

Plasma miRNA-210ΔCt, miRNA-373 and let-7 expressions were compared between patients with and without comorbid conditions. Although expression of miR-NA-210ΔCt was up-regulated in patients with comorbid conditions and, miRNA-373ΔCt and let-7ΔCt expressions were down-regulated, these differences were not statistically significant. (p=0.22; p=0.65; and p=0.10 respectively). Statistically, up-regulated miRNA-210ΔCt expression in IDA was not affected by the comorbidities (Table 3).

**Table 3:** Relation of miRNA and co-morbidities

		N	Mean±SD	*p* value
**MiRNA-210ΔCt**	**No co-morbidity**	15	-6,96±2.46	0.022
	**Co-morbidity**	20	-7,52±2,08	
**MiRNA-373ΔCt**	**No co-morbidity**	15	-7.71±2,08	0.65
	**Co-morbidity**	20	-7,09±2,93	
		**N**	**Median±ÇAA**	***p* value**
**Let-7ΔCt**	**No co-morbidity**	15	3,13±2,18	0.10
	**Co-morbidity**	20	2,25±1,81	

The independent effects of miRNA expression on the development of iron deficiency were evaluated by logistic regression analysis. Changes in expression were found to be independent risk factors that increase the risk of developing iron deficiency. When the patients were evaluated compared to the control group, let-7 exchange per unit led to the 1,15-fold of having IDA. For miRNA-373 exchange per unit, IDA risk increased by 1.01-fold and with of miRNA-210, IDA risk decreased by 1,25-fold (p>0.05) (Table 4).

**Table 4:** The effect of miRNAs on the development of IDA

	Exp(B)	95% C.I.for EXP(B)		*p*
		lower	upper	
**MiRNA-210 dct**	1,25	0,79	1,97	0,33
**MiRNA-373 dct**	1,01	0,72	1,42	0,91
**Let-7dct**	1,15	0,64	2,07	0,62

## Discussion

Many studies have shown that IDA may be associated with many chronic diseases such as chronic kidney disease, chronic heart failure, inflammatory bowel disease and cancer 16-18. Yalcin et al showed that the thiol / disulphide balance was deteriorated towards the oxidant side in IDA patients compared to control group, and there could be oxidative damage 19. Binding and depletion of iron present in vascular smooth muscle cells decreased the number of cells undergoing apoptosis. These findings suggest that iron is an essential element for cell death 20.

MiRNAs also play a role in iron homeostasis, which post-transcriptionally regulates genes associated with cellular iron uptake, storage, and utilization. When there is not enough iron, hemoglobin production decreases, oxygen carrying capacity of blood decreases and tissue oxygen transmission is limited. Iron stores are used in the presence of limited iron availability for maintenance of iron homeostasis. When oxygen levels are low, iron stores are mobilized to restore oxygen availability in tissues and increase hemoglobin and erythrocyte production 21-22.

Biogenesis and expression of miRNAs are largely regulated by hypoxia. After hypoxia, it has been shown that miR-210 and miR-373 actively reduced the gene expressions responsible for DNA repair 21. Hypoxia has an important place in IDA; in fact, it was speculated that the expression of genes responsible for DNA repair might be decreased in IDA due to hypoxia. It was shown that, let-7, which is an important tumour suppressor, decreased after oxidative stress 15. Crosby et al. highlighted that hypoxic stress induced a series of miRNA expression in human cells. One of these, miR-210, was predicted to target the mRNA of the DNA repair gene RAD52, while miR-373 targets both RAD52 and RAD23B. In the analysis, it was observed that overexpression of miR-210 or miR-373 decreased RAD52 levels and miR-373 suppressed RAD23B expression levels 15, 21. RAD52 is a defining member of a group of genes involved in the repair of DNA double strand breaks caused by ionizing radiation. The regulation of these RAD52 activities as well as their subnuclear localization is important for ensuring the regular and correct repair many of the endogenous and exogenous DNA lesions 23. RAD23 is a nucleotide-excision repair protein and plays an important role in DNA repair and stabilization of stress factors 24. It has been shown that miR-210 expression increased in both normal and cancerous cells in a hypoxic environment 25, 26.

MiR-210 had been also/span> effective on proliferation and apoptosis and high levels of miR-210 had proliferative and anti-apoptotic effects on Glioblastoma multiforme cell lines 27, 28. Interestingly, while miR-210 was pro-apoptotic in non-hypoxic environment, it exhibited anti-apoptotic properties in hypoxia 29. In addition, expression of miR-210 is increased during red cell maturation process 30. In a study in which patients with beta thalassemia and HgE were included, miR-210 level was found to be increased in both serum and erythrocytes, and miR-210 level was found to be negatively correlated with the hemoglobin level 31.

In our study, plasma miR-210 expression was found significantly upregulated in patients compared to healthy group (p = 0.022). High levels of miR-210 in the anemia are compatible with the literature. If a patient with IDA doesn't receive treatment for a long time for any reason, it may lead to chronic miR-210 elevation in hypoxic environment. In this case, it is theoretically possible that, miR-210 may exert anti-apoptotic effects and cell proliferation.

MiR-373 has profound effects on cancer but studies yielded conflicting results 32. In the case of hypoxia, hypoxia induced factor 1 upregulated the levels of miR-373 expression 21. It was found to be increased 1530 times more than normal in seminoma cell culture compared to normal testicular tissue 33. In a cell culture study, there was a significant positive correlation between miR-373 levels and breast cancer cell migration and invasion 34. In a study conducted in a cell culture capable of secreting endogenous miR-373, there was a significant positive correlation between miR-373 expression and migration and invasion capabilities of cancer cells 35. In contrast, elevated levels of miR-373 significantly reduced migration and invasion in breast cancer cell culture and miR-373 level was found to be higher in breast cancer patients with lymph node metastasis compared to those without 36, 37. Also, expression of miR-373 was elevated in breast cancer patients compared to healthy people and its expression was found to be significantly increased in HER2 negative breast cancer patients compared to HER2 positive patients 34.

The elevation of miR-373 has been related to poor prognosis in liver cancers 38. MiR-373 expression has been found to be elevated significantly in tumour tissues and miR-373 was highlighted as an oncogene 39, 40. However, decreased miR-373 expression levels were also observed in both tumour tissues and cancer cell lines in prostate cancer, and exogenous miR-373 did not stop the growth of the tumour, on the contrary, it was observed to accelerate migration and invasion by inhibiting CD44 translation 41. In our study, it was found that plasma miR-373 expression was decreased in the patient group compared to controls although no significant difference was found (p = 0.10). The reduction of miR-373 may also be a defence mechanism to prevent cancer formation, as in prostate cancer, considering pro-oncogene changes in other miRNAs.

Let-7 is a miRNA that shows tumour suppressor activity in mammals 42. Let-7 has been shown to inhibit the cell cycle regulators cyclin D1, cyclin D3, cyclin A and CDK4 and stop tumour growth 43. In cell culture studies, Let-7 has shown an inhibitory effect on Burkitt lymphoma cell lines 44. It has been also shown to be high in mammals, in embryogenesis and during brain formation 45. In addition, studies have shown that let-7 regulates hematopoietic stem cells 46. The suppression of let-7 was found to be positively correlated with suppression of growth in red cells 47. However, let-7's activities in mammals are still not fully known. In our study, down-regulation in expression of let-7 was observed in patients with IDA, but it was not statistically significant; this may be related to the small size of our working group. Down-regulation of Let-7 was frequently observed in malignancies and was mostly associated with poor prognosis, and it was interesting to reach the same conclusion in IDA 48. In addition, while erythropoiesis is induced with miR-210 elevation, it is also suppressed with down-regulation of let-7 so; these two miRNAs show an antagonistic effect on erythropoiesis.

The alterations of miRNA expression were also shown to be significant in our study. Each unit change of let-7 and miR-373 had led to 4.23 and 1.01-fold more of having IDA respectively. Conversely, each unit of change on expression of miR-210 was correlated with decreased the risk of having IDA. These results clearly showed the link between IDA and miRNAs.

There were some limitations in our study; the number of participants is low to interpret broader suggestions about relation between cancer formation and IDA. The study design was retrospective and cross-sectional; a larger study group is clearly needed to test our results. Also, the study group included female patients; high prevalence of IDA in women had led the authors to choose women as the study group. Oncogenesis is a multifactorial process, changes only in miRNA levels may not led to cancer process; so direct and definite conclusions should not be made upon only alteration of miRNAs. It must be also defined whether; iron therapy would restore miRNA's level to normal. It is also not ethical to follow-up patients without restoring iron stores to normal but, many patients in low-income countries have been suffering chronic iron deficiency and those patients could be screened for malignancies to get a definite link between IDA and miRNAs. In addition, our study sample was younger and healthier than a normal population study group; we excluded acute or chronic infections, chronic inflammatory disease and patients with active cancer which could make impossible to reach conclusions about general population. The main strength of our study is that it is the first in its field to evaluate the relationship between IDA and malignancy-related miRNAs so the significant data would clearly add new and valuable information to the literature and should led to new studies on this field.

In summary, plasma miR-210 expression was significantly upregulated, and miR-373 and let-7 expression was downregulated in the IDA group compared to the control group which were also found to be affected in malignancies. It will be valuable to investigate whether there is an increase in incidence of malignancies and whether treatment of IDA would restore miRNAs to normal, by conducting studies in patients with chronic untreated IDA.
